# High Expression Level of Tra2-β1 Is Responsible for Increased *SMN2* Exon 7 Inclusion in the Testis of SMA Mice

**DOI:** 10.1371/journal.pone.0120721

**Published:** 2015-03-17

**Authors:** Yu-Chia Chen, Jan-Gowth Chang, Yuh-Jyh Jong, Ting-Yuan Liu, Chung-Yee Yuo

**Affiliations:** 1 Graduate Institute of Medicine, Kaohsiung Medical University, Kaohsiung, Taiwan; 2 Epigenome Research Center, China Medical University Hospital, Taichung, Taiwan; 3 Department of Laboratory Medicine, China Medical University Hospital, Taichung, Taiwan; 4 School of Medicine, China Medical University, Taichung, Taiwan; 5 Departments of Pediatrics and Clinical Laboratory, Kaohsiung Medical University Hospital, Kaohsiung Medical University, Kaohsiung, Taiwan; 6 Department of Biological Science and Technology, National Chiao Tung University, Hsinchu, Taiwan; 7 Department of Biomedical Science and Environmental Biology, Kaohsiung Medical University, Kaohsiung, Taiwan; University of Edinburgh, UNITED KINGDOM

## Abstract

Spinal muscular atrophy (SMA) is an inherited neuromuscular disease caused by deletion or mutation of *SMN1* gene. All SMA patients carry a nearly identical *SMN2* gene, which produces low level of SMN protein due to mRNA exon 7 exclusion. Previously, we found that the testis of SMA mice (*smn^−/−^ SMN2*) expresses high level of *SMN2* full-length mRNA, indicating a testis-specific mechanism for *SMN2* exon 7 inclusion. To elucidate the underlying mechanism, we established primary cultures of testis cells from SMA mice and analyzed them for *SMN2* exon 7 splicing. We found that primary testis cells after a 2-hour culture still expressed high level of *SMN2* full-length mRNA, but the level decreased after longer cultures. We then compared the protein levels of relevant splicing factors, and found that the level of Tra2-β1 also decreased during testis cell culture, correlated with *SMN2* full-length mRNA downregulation. In addition, the testis of SMA mice expressed the highest level of Tra2-β1 among the many tissues examined. Furthermore, overexpression of Tra2-β1, but not ASF/SF2, increased *SMN2* minigene exon 7 inclusion in primary testis cells and spinal cord neurons, whereas knockdown of Tra2-β1 decreased *SMN2* exon 7 inclusion in primary testis cells of SMA mice. Therefore, our results indicate that high expression level of Tra2-β1 is responsible for increased *SMN2* exon 7 inclusion in the testis of SMA mice. This study also suggests that the expression level of Tra2-β1 may be a modifying factor of SMA disease and a potential target for SMA treatment.

## Introduction

Spinal muscular atrophy (SMA) is an autosomal recessive disease characterized by the loss of lower motor neurons and atrophy of muscles. According to age of onset and its severity, SMA is categorized into three main types (type I—III) and two subtypes (type 0 and type IV) [1,2]. SMA occurs in approximately 1 in 6,000 to 10,000 newborns and has a carrier frequency of 1 in 35 to 50. SMA is the second most common autosomal recessive disease and the most common genetic cause of infant mortality [3].

SMA is caused by deletions or mutations of *survival motor neuron 1* (*SMN1*) gene. All SMA patients carry a nearly identical *SMN2* gene. However, *SMN2* expresses only limited amount of functional SMN protein and is unable to compensate for the *SMN1* gene defect [4]. The two genes differ by only five nucleotides, and the most important difference between *SMN1* gene and *SMN2* gene is a C-to-T transition at 6^th^ nucleotide of exon 7. The nucleotide transition causes exon 7 exclusion in most *SMN2* mRNA [5–8].

According to previous studies, there are two putative splicing models of *SMN1* and *SMN2* mRNAs: the C-to-U change in *SMN2* mRNA causes the loss of a serine/arginine-rich (SR) splicing factor (SF2/ASF)–dependent exonic splicing enhancer (ESE) [9] or the creation of a heterogeneous nuclear ribonucleoprotein (hnRNP) A1–dependent exonic splicing silencer (ESS) [10]. In the central part of exon 7, an AG-rich ESE is recognized by the SR-like splicing factor Htra2-β1 which can promote exon 7 inclusion [11]. The Htra2-β1 also serves as a nucleation point for several additional regulatory factors that indirectly associate with *SMN* exon 7, including SRp30c and hnRNP-G [12,13].

The SMN protein is complexed with several proteins, including Gemins 2–8 and Unr-interacting protein (UNRIP) [14–22]. The SMN complex has multiple functions. One of the most important functions of the SMN complex is as an assemblyosome that is necessary for biogenesis of spliceosomal small nuclear ribonucleoproteins (snRNPs) [23–26]. The snRNP complexes are known as the major components of spliceosome. The major spliceosome consists of the snRNPs U1, U2, U4/U6, and U5, which is responsible for most pre-mRNA splicing. The minor spliceosome, by contrast, comprises snRNPs U11, U12, U4atac/U6atac, and U5, which is responsible for the splicing of a rare class of introns that have non-canonical consensus sequences [27]. The SMN complex also functions as mRNA carrier that promotes β-actin mRNA transport to the axon of motor neurons. The neuron-specific function might be the reason that the ubiquitously-expressed SMN protein defection causes motor neuron degeneration [25,28,29].

The first SMA-like mouse models were developed independently at the same time by Li group and Burghes group [30,31]. Mice have only one *smn* gene, which has similar function with human *SMN1*. Knockout of *smn* gene in mice caused embryonic lethal before E6.5. The SMA-like mice were generated by crossing *smn* ^+/−^ mice with human *SMN2* transgene, which showed motor neuron degenerative symptoms [30]. Our previous study showed that the testis expressed high levels of *SMN2* full-length mRNA and SMN protein, which is different from other tissues of SMA mice. In addition, hnRNP Q, a *SMN* exon 7-interacting protein, was found to be expressed at higher level in the testis compared with brain and liver. Overexpression of hnRNP Q1 promoted *SMN2* exon 7 inclusion, but hnRNP Q2/3 had a negative role in *SMN* mRNA splicing. Therefore, differential expression of hnRNP Q isoforms might intricately regulate *SMN* mRNA splicing [32]. To further investigate the mechanism of *SMN2* mRNA splicing in the testis of SMA mice, we established the primary testis cell culture from SMA mice. We found that the *SMN2* full-length mRNA decreased in 96 hour-cultured primary testis cells compared with the testis tissue and 2 hour-cultured cells. Among the splicing factors we examined in primary testis cells, we found the expression of Tra2-β1 also dramatically decreased in 96 hour-cultured cells compared with 2 hour-cultured cells. In contrast, the expression levels of hnRNP Q showed no significant difference in primary testis cells of SMA mice. In addition, overexpression of Tra2-β1 in the primary testis cells increased *SMN2* full-length mRNA expression, suggesting that *SMN2* mRNA splicing change in testis cells is regulated by Tra2-β1.

## Materials and Methods

### Animals

18–20-week-old type 3 SMA mice were used for the experiments. The mice were sacrificed by high concentration of carbon dioxide and the tissues were harvested after sacrifice. All breeding and subsequent use of animals, including mice sacrifice, in this study were approved by the Institutional Animal Care and Use Committee of Kaohsiung Medical University. The IACUC approval number is 98033.

### Primary culture of testis cells from SMA mice

The testes were harvested from adult type 3 SMA mice (*smn* ^*−/−*^ *SMN2* ^*+*^) [30] and decapsulated in PBS. Then the tissues were incubated with type IV collagenase (2.5 mg/ml) for 30 minutes at 37°C in serum-free culture medium (DMEM/F12) supplemented with 15 mM HEPES. The tissues and cells were centrifuged (5 minutes, 400g) and resuspended in 2 ml of Hank’s balanced salt solution containing 0.1% Trypsin-EDTA and 40 unit DNaseⅠ and incubated for 10 minutes at 37°C. After centrifugation at 400g, the cells were washed 5 times by PBS and then resuspended in culture medium containing 5% horse serum and 2.5% fetal bovine serum. The cells were seeding on 0.1% gelatin-coated dishes and incubated at 37°C with 5% CO_2_.

### Primary culture of spinal neuron cells from SMA mouse embryos

The spinal cord were harvested from E17.5 embryos of SMA mice (*smn* ^*−/−*^ *SMN2* ^*+*^). Six to eight embryos from the same litter were collected together. The spinal cord were digested with 0.025% Trypsin-EDTA in GHEBS buffer (137 mM NaCl, 2.7 mM KCl, 22.2 mM glucose, 25 mM HEPES, pH 7.4, and 20 U/ml penicillin plus 20 mg/ml streptomycin) for 10 minutes at 37°C. Then the tissues were homogenized by mechanical dissociation and moved to 4% BSA in GHEBS buffer. The cells were centrifuged (10 minutes, 520g) and resuspended in 1 ml GHEBS buffer. The cells were isolated by centrifugation (10 minutes, 520g) on an Iodixanol (OptiPrep, Axis-Shield) gradient solution. Iodixanol solution (11.5%) was freshly prepared in GHEBS buffer. A sharp band on the top of the Iodixanol solution was the neuron fraction. The fraction was collected and resuspended in GHEBS buffer. The cells were centrifuged (10 minutes, 520g) in 4% BSA again and resuspended in culture medium (Neurobasal supplemented with B-27) containing 2% horse serum, 0.5 mM L-glutamine, and 25 μM 2-mercaptoethanol). The cells were seeded in 6-well plates and incubated at 37°C with 5% CO_2_ for 3 days. The plates were coated with poly-ornithine (30 μg/ml) for 30 minutes and laminin (2 μg/ml) for 60 minutes.

### Cell culture and transfection

Mouse testis cell lines, GC-1 spermatogonia (from Bioresource Collection and Research Center; BCRC Number: 60312) were grown in Dulbecco's Modified Eagle Medium with 4 mM L-glutamine adjusted to contain 1.5 g/L sodium bicarbonate, 4.5 g/L glucose, 1.0 mM sodium pyruvate, and 10% fetal bovine serum at 37°C in a humidified atmosphere containing 5% CO_2_. TM3 Leydig cells (from Bioresource Collection and Research Center; BCRC Number: 60475) and TM4 Sertoli cells (from Bioresource Collection and Research Center; BCRC Number: 60254) were grown in Dulbecco's Modified Eagle Medium/F12 with 4.5 g/L glucose, 2.5 mM L-glutamine, 0.5 mM sodium pyruvate, 1.2 g/L sodium bicarbonate, 15 mM HEPES supplemented with 5% horse serum and 2.5% fetal bovine serum at 37°C containing 5% CO_2_. The mouse testis cell lines, primary testis cells and primary neuron cells were transfected with pIRES-*SMN2* minigene-luc plasmid (provided by Dr. Minlei Zhang [33]) by Lipofectamine 2000 Transfection Reagent (Invitrogen) according to manufacturer’s instructions. The primary testis cells were transfected with Tra2-β1 siRNA (Ambion, siRNA ID: s73772) by Lipofectamine 2000 Transfection Reagent. After 48 hours of transfection, total RNA was extracted from transfected cells.

### RNA extraction, RT-PCR, and quantitative RT-PCR

Total RNA was isolated from tissues or cells by TRIzol Reagent (Invitrogen). One microgram RNA was reverse-transcribed into cDNA by using SuperScript III Reverse Transcriptase kit (Invitrogen). The PCR primers: *SMN2* forward, 5’-CCC ACC ACC TCC CAT ATG TCC-3’, and *SMN2* reverse, 5’-AAC TGC CTC ACC ACC GTG CTG-3’; *SMN2* minigene forward, 5’-CCA CCA CCT CCC ATA TGT CCA-3’, and *SMN2* minigene reverse, 5’-AGC TTC TGC CAA CCG AAC GGA-3’. The *SMN2* PCR reaction was carried out as follows: 94°C for 5 min, then 35 cycles of 94°C for 30 sec, 60°C for 45 sec, 72°C for 1 min, and final extension at 72°C for 7 min. The *SMN2* minigene PCR reaction was the same as *SMN2* gene, except that the annealing temperature was 55°C. The PCR product derived from exon 7–containing *SMN2* mRNA (421bp) and that derived from exon 7–lacking *SMN2* mRNA (367bp) were separated by 3% agarose gel electrophoresis. The sizes of *SMN2* minigene PCR products were 375 bp and 322 bp. The intensity of the PCR products was analyzed by LabWorks Image Acquisition and Analysis Software (UVP BioImaging Systems). qRT-PCR was performed on Roche LightCycler 480 Real-Time PCR System using Universal ProbeLibrary System (Roche). The primers for real-time PCR: *Tra2-β1* forward, 5’-GGG TTA TGA TGA CCG GGA CT-3’, and *Tra2-β1* reverse: 5’- TCT GAT CCC TGT CTT GAG CTG-3’; *ASF/SF2* forward, 5’-TCC GAG AAC AGA GTG GT TGT C-3’, and *ASF/SF2* reverse, 5’-CAT ACA TCA CCT GCC TCA CG-3’. The universal probe for *Tra2-β1* is #58 (Roche, cat. no. 04688554001) and #16 (Roche, cat. no. 04686896001) for *ASF/SF2*.

### Protein extraction and Western blot

The proteins were extracted from tissues or cells using RIPA lysis buffer (50 mM Tris, pH8.0, 150 mM NaCl, 1 mM EDTA, 1% NP40, 1% sodium deoxycholate, 0.1% SDS) and analyzed in SDS-PAGE as the following description: after electrophoresis, the proteins were transferred onto nitrocellulose membrane (GE Healthcare). The membranes were blocked with 5% skim milk solution in TBST for one hour. The membranes were exposed to the primary antibody in 1% skim milk solution for one hour before they were washed three times for ten minutes with TBST, and then incubated for one hour with HRP-conjugated secondary antibody. After washing three times with TBST, the membranes were immersed in the ECL plus substrate (Amersham) for five minutes. The chemiluminescence signals were detected by X-ray films and analyzed by LabWorks Image Analysis Software. The primary antibodies used were anti-SMN (BD; 1:5000 dilution), anti-TRA2B (Abcam; 1:1000 dilution), anti-SF2/ASF (Zymed Laboratories Inc.; 1:1000 dilution), anti-hnRNP A1 (9H10, SIGMA; 1:1000 dilution), anti-hnRNP Q (Abcam; 1:1000 dilution), anti-SFRS9 (Abcam; 1:1000 dilution), and anti-Actin (Santa Cruz Biotechnology, Inc.; 1:3000 dilution). The secondary antibodies used were horse anti-mouse IgG-HRP (Cell Signaling; 1:5000 dilution), and donkey anti-goat IgG-HRP (Santa Cruz Biotechnology, Inc.; 1:5000 dilution).

### snRNP complex assembly

The cell lysate was extracted from primary testis cells using RSB-100 buffer (100 mM NaCl, 10 mM Tris-HCl, pH7.4, 2.5 mM MgCl_2_, and 0.1% NP-40) containing protease inhibitor cocktail (Roche) and phosphatase inhibitors (50 mM NaF, 0.2 mM Na_3_VO_4_). After pre-cleaning with protein-G agarose for 1 h at 4°C, immunoprecipitations with anti-Sm (Smith Antigen) antibodies (Y12, Thermo Scientific) from cell extracts (30 μg) were carried out in RSB-500 buffer (500 mM NaCl, 10 mM Tris-HCl, pH 7.4, 2.5 mM MgCl_2_ and 0.1% NP-40) containing protease inhibitor cocktail and phosphatase inhibitors for 2 h at 4°C. After five washes with the same buffer, bound RNAs were isolated from immunoprecipitates by TRIzol Reagent extraction and ethanol precipitation. snRNA-specific reverse primers were used to generate cDNA using the SuperScript III Reverse Transcriptase kit. Quantification of snRNAs was carried out by real-time PCR experiments on Applied Biosystems 7500 fast real-time PCR system using SYBR Green. The same primers were used in both reverse transcription and real-time PCR. The primers used were: U1 forward, 5’-GAT ACC ATG ATC ACG AAG GTG GTT-3’, U1 reverse, 5’-CAC AAA TTA TGC AGT CGA GTT TCC-3’; U2 forward, 5’-TTT GGC TAA GAT CAA GTG TAG TAT CTG TTC-3’, U2 reverse, 5’-AAT CCA TTT AAT ATA TTG TCC TCG GAT AGA-3’; U4 forward, 5’-GCG CGA TTA TTG CTA ATT GAA A-3’,U4 reverse, 5’-AAA AAT TGC CAA TGC CGA CTA-3’; U5 forward, 5’-TAC TCT GGT TTC TCT TCA GAT CGT ATA AAT-3’, U5 reverse, 5’-AAT TGG TTT AAG ACT CAG AGT TGT TCC T-3’; U6 forward, 5’-GCT TCG GCA GCA CAT ATA CTA AAA T-3’, U6 reverse, 5’-ACG AAT TTG CGT GTC ATC CTT-3’.

### Statistical analysis

Statistical analysis of data was performed using Microsoft Excel 2010 software (Microsoft, Redmond, WA). Student’s *t* test was conducted to evaluate differences between groups. A probability of less than 0.05 was considered to be statistically significant.

## Results

### The testis of SMA mice expresses high level of *SMN2* full-length mRNA and SMN protein

**Fig 1:**
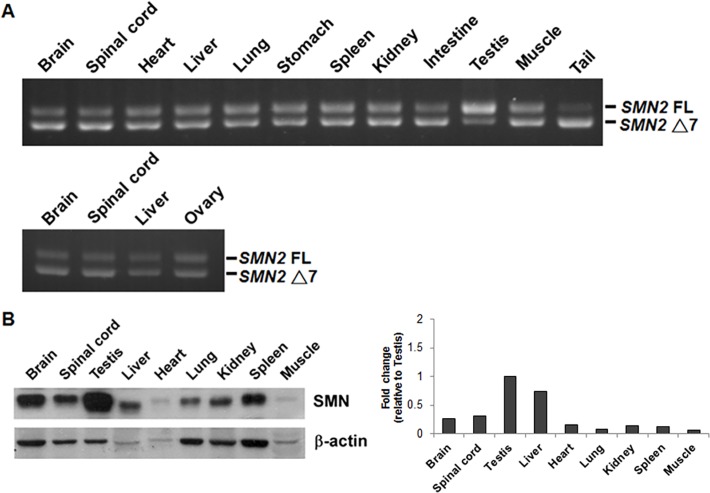
The testis of SMA mice expresses high levels of *SMN2* full-length mRNA and protein. Various tissues of type 3 SMA mice were collected. (A) Total RNA was isolated and subjected to RT-PCR to amplify both *SMN2* full-length (FL) and exon 7-lacking (Δ7) mRNAs. A representative result of six independent experiments was shown. The result showed that the testis expressed high level of *SMN2* FL mRNA. (B) Proteins were extracted and subjected to Western blotting to detect SMN protein expression. The result showed that SMA mouse testis expressed the highest level of SMN protein among the tissues examined.

The SMA mice we used are deficient for mouse *smn* and express human *SMN2* [30] and show pathological changes in the spinal cord and skeletal muscles similar to those of SMA patients. We examined the *SMN2* mRNA splicing and SMN protein expression in several tissues of type 3 SMA mice, including brain, spinal cord, heart, liver, lung, stomach, kidney, spleen, intestine, muscle, tail, testis in male mice, and ovary in female mice. Among the tissues we examined, we found that the testis expressed high level of *SMN2* full-length (FL) mRNA (Fig. 1A and see S1 Fig. for qRT-PCR result) and SMN protein (Fig. 1B). According to the result, the ovary of SMA mice expressed high level of *SMN2* truncated mRNA (Fig. 1A), indicating that the special splicing pattern in the testis is not germ cell-related. Thus, our results indicate a testis-specific mechanism of *SMN2* mRNA splicing in SMA mice.

### The levels of *SMN2* full-length mRNA and protein decrease during testis cell primary culture

To investigate the testis-specific mechanism of *SMN2* mRNA splicing, we detected *SMN2* mRNA splicing in three different mouse testis cell lines, including GC-1, TM3 and TM4, with *SMN2* minigene transfection. GC-1 spermatogonia, TM3 Leydig cells and TM4 Sertoli cells are three major cell types in the testis. The result showed that *SMN2* exon 7 inclusion is not promoted in mouse testis cell lines (S2 Fig.). We supposed that the special *SMN2* mRNA splicing pattern in the testis is physiologically dependent. Therefore, ex vivo cultured mouse testis cell lines were unable to express high level of *SMN2* full-length mRNA.

**Fig 2:**
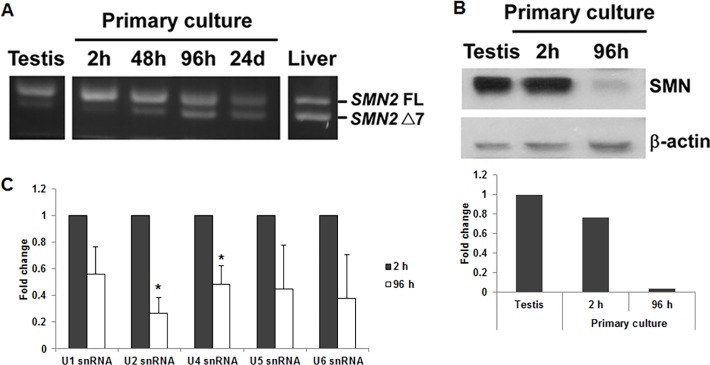
The levels of *SMN2* full-length mRNA and protein decrease during testis cell primary culture. (A) Total RNA was isolated from primary testis cells cultured for different time periods (2 hours, 48 hours, 96 hours and 24 days) and subjected to RT-PCR to amplify *SMN2* FL and Δ7 mRNAs. For comparison, total RNA isolated directly from the testis and liver was also analyzed. A representative result of three independent experiments was shown. The result showed that primary testis cells after a 2-hour culture still expressed high level of *SMN2* FL mRNA. However, the level decreased after longer cultures. (B) Proteins extracted from primary testis cells cultured for 2 hours and 96 hours were subjected to Western blotting to detect SMN protein. The result showed that SMN protein level was high in 2-hour cultured cells and decreased dramatically in 96-hour cultured cells, consistent with the result of *SMN2* exon 7 splicing. (C) snRNP complexes were isolated from primary testis cells cultured for 2 hours and 96 hours by anti-Sm antibodies. Various snRNAs were then extracted and quantitated by real-time PCR. The result showed that the levels of snRNP complexes decreased in 96-hour cultured cells compared with 2-hour cultured cells. Error bars represent standard deviation. (*) *P* < 0.05, compared with 2-hour cultured cells.

To solve the problem, we established primary cultures of testis cells from SMA mice and analyzed them for *SMN2* exon 7 splicing. We found that SMA primary testis cells after a 2-hour culture still expressed high level of *SMN2* full-length mRNA. However, the level decreased after longer cultures (Fig. 2A and see S3 Fig. for qRT-PCR result). In addition, SMN protein level was high in 2-hour cultured cells and decreased dramatically in 96-hour cultured cells, consistent with the result of *SMN2* exon 7 splicing (Fig. 2B). We also explored the function of SMN protein by measuring snRNP complex assembly. According to previous studies, SMN protein is part of the SMN complex and one of the functions of SMN complex is to participate in snRNP assembly [34]. The result showed that snRNP assembly decreased in 96-hour cultured cells compared with 2-hour cultured cells (Fig. 2C), indicating that decreased SMN expression was accompanied with the decrease of SMN functions. Therefore, our results showed that the levels of *SMN2* full-length mRNA and protein decreased during testis cell primary culture, indicating the factor that promotes *SMN2* exon 7 inclusion in the testis gradually disappears in the cell culture system.

### The levels of Tra2-β1 and ASF/SF2 decrease during testis cell primary culture, which are correlated with *SMN2* exon 7 splicing

**Fig 3:**
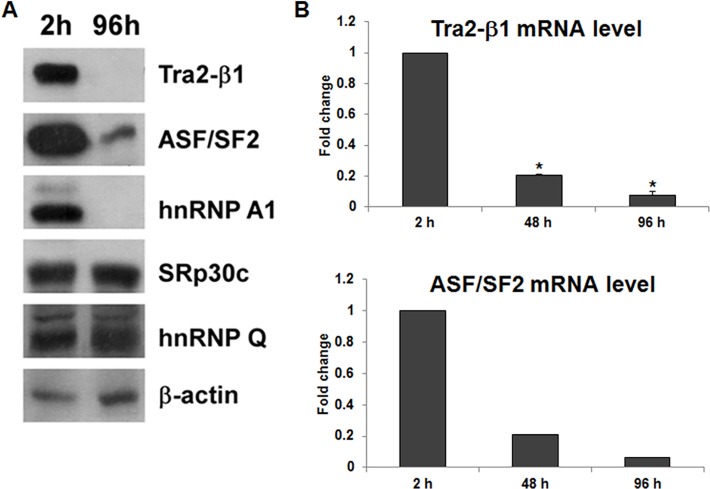
The levels of Tra2-β1 and ASF/SF2 decrease during testis cell primary culture, which is correlated with *SMN2* exon 7 splicing. (A) Proteins extracted from primary testis cells cultured for 2 hours and 96 hours were subjected to Western blotting to detect Tra2-β1, ASF/SF2, hnRNP A1, SRp30c, and hnRNP Q. A representative result of two independent experiments was shown. The result showed that the levels of Tra2-β1, ASF/SF2, and hnRNP A1 decreased dramatically from 2-hour to 96-hour cultures. (B) Total RNA was isolated from primary testis cells cultured for different time periods and subjected to qRT-PCR to detect *Tra2-β1* and *ASF/SF2* mRNA expression levels. The result showed that the mRNA levels of *Tra2-β1* and *ASF/SF2* decreased after longer cultures, which was consistent with the protein expression. Error bars represent standard deviation. (*) *P* < 0.01, compared with 2-hour cultured cells.

To investigate the mechanism of *SMN2* full-length mRNA downregulation in the primary culture cell system, we compared the protein levels of relevant splicing factors, including ASF/SF2, hnRNP A1, Tra2-β1, SRp30c, and hnRNP Q, in 2-hour and 96-hour cultured cells. The protein levels of Tra2-β1 and ASF/SF2 were high in 2-hour cultured cells and decreased dramatically after 96 hours (Fig. 3A), consistent with the result of *SMN2* exon 7 splicing. The mRNA levels of *Tra2-β1* and *ASF/SF2* also decreased gradually during testis cell primary culture (Fig. 3B). We also observed that the protein level of hnRNP A1 was low in 96-hour cultures cells compared with 2-hour cultured cells (Fig. 3A). However, previous studies showed that hnRNP A1 binds to the splicing silencer in *SMN2* exon7 and acts as a splicing repressor to cause *SMN2* exon 7 exclusion [10,35]. Thus, the downregulation of hnRNP A1 cannot explain the result of increased *SMN2* exon 7 exclusion. Therefore, our results show that the changes in the levels of Tra2-β1 and ASF/SF2 during testis cell primary culture are correlated with *SMN2* full-length mRNA downregulation.

### The testis expresses the highest level of Tra2-β1 among the tissues examined

**Fig 4:**
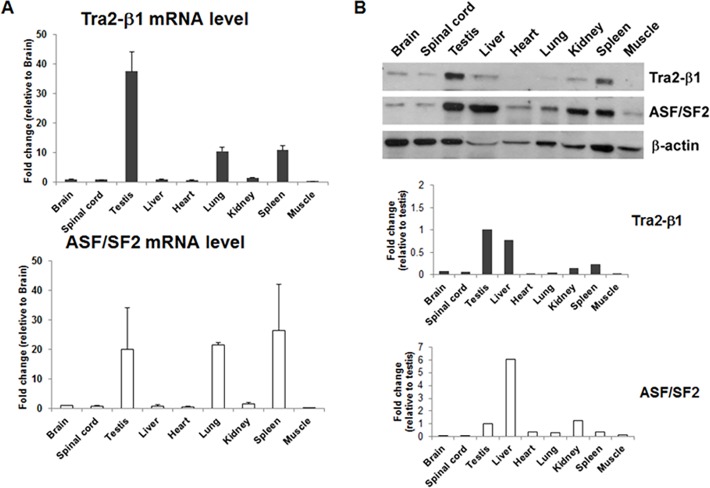
The testis expresses the highest level of Tra2-β1 among the tissues examined. Various tissues of 18–20-week-old type 3 SMA mice were collected. (A) Total RNA was isolated and subjected to qRT-PCR to detect *Tra2-β1* and *ASF/SF2* mRNA levels. The result showed that among the tissues examined, the testis expressed the highest level of *Tra2-β1* mRNA, but not the highest level of *ASF/SF2* mRNA. (B) Proteins were extracted and subjected to Western blotting to detect Tra2-β1 and ASF/SF2 proteins. The result also showed that among the tissues examined, the testis expressed the highest level of Tra2-β1 protein, but not the highest level of ASF/SF2 protein.

To confirm the importance of Tra2-β1 and ASF/SF2 in testis-specific *SMN2* splicing, we examined mRNA and protein levels of Tra2-β1 and ASF/SF2 in several tissues of type 3 SMA mice. The result showed that the testis expressed the highest level of Tra2-β1 mRNA and protein (Fig. 4A and 4B). In contrast, the expression levels of ASF/SF2 mRNA and protein were not the highest in the testis (Fig. 4A and 4B). The results support that the high level of Tra2-β1 in the testis plays an important role in *SMN2* exon 7 inclusion.

### Overexpression of Tra2-β1, but not ASF/SF2, increases *SMN2* exon 7 inclusion in primary testis cells and spinal cord neurons of SMA mice

**Fig 5:**
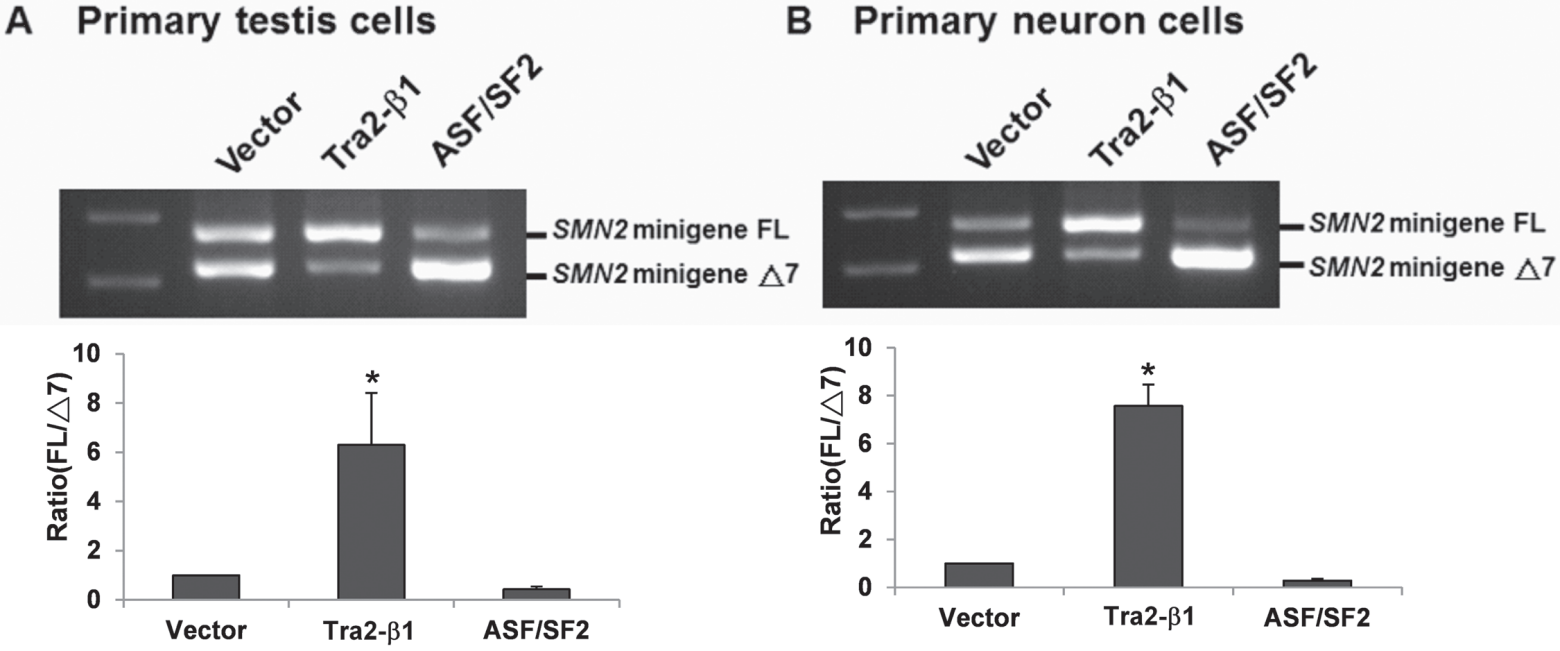
Overexpression of Tra2-β1, but not ASF/SF2, increases *SMN2* exon 7 inclusion in primary testis cells and spinal cord neurons of SMA mice. Primary testis cells (A) and primary spinal cord neurons (B) of SMA mice were co-transfected with *SMN2* minigene plasmid and *Tra2-β1* overexpression plasmid, *ASF/SF2* overexpression plasmid or blank vector as control for 48 hours. Total RNA was isolated from transfected cells and then subjected to RT-PCR to amplify *SMN2* minigene FL and Δ7 mRNAs. The result showed that overexpression of Tra2-β1, but not ASF/SF2, remarkably increased *SMN2* exon 7 inclusion in both primary testis cells and primary spinal cord neurons of SMA mice. Error bars represent standard deviation. (*) *P* < 0.05, compared with the vector control.

To clarify whether Tra2-β1 contributes to *SMN2* exon 7 inclusion in the testis, we overexpressed Tra2-β1 or ASF/SF2 in 96-hour cultured primary testis cells. Because of the low transfection efficiency, the testis primary cells were transfected with overexpression plasmids and *SMN2* minigene plasmid. Then we evaluated the effect of Tra2-β1 or ASF/SF2 overexpression by detecting *SMN2* minigene splicing. The result showed that overexpression of Tra2-β1 enhanced *SMN2* minigene exon 7 inclusion (Fig. 5A). However, overexpression of ASF/SF2 had no effect on *SMN2* minigene exon 7 inclusion (Fig. 5A), indicating Tra2-β1 is the factor that plays a crucial role in *SMN2* exon 7 inclusion in the testis of SMA mice. To further investigate the effect of Tra2-β1 in neuron cells of SMA mice, we also established primary neurons from spinal cords of SMA mouse embryos and overexpressed Tra2-β1 or ASF/SF2 in primary neurons. The result showed that overexpression of Tra2-β1, but not ASF/SF2, also enhanced *SMN2* minigene exon 7 inclusion in spinal cord neurons of SMA mice (Fig. 5B).

### Knockdown of Tra2-β1 decreases *SMN2* exon 7 inclusion in primary testis cells of SMA mice

**Fig 6:**
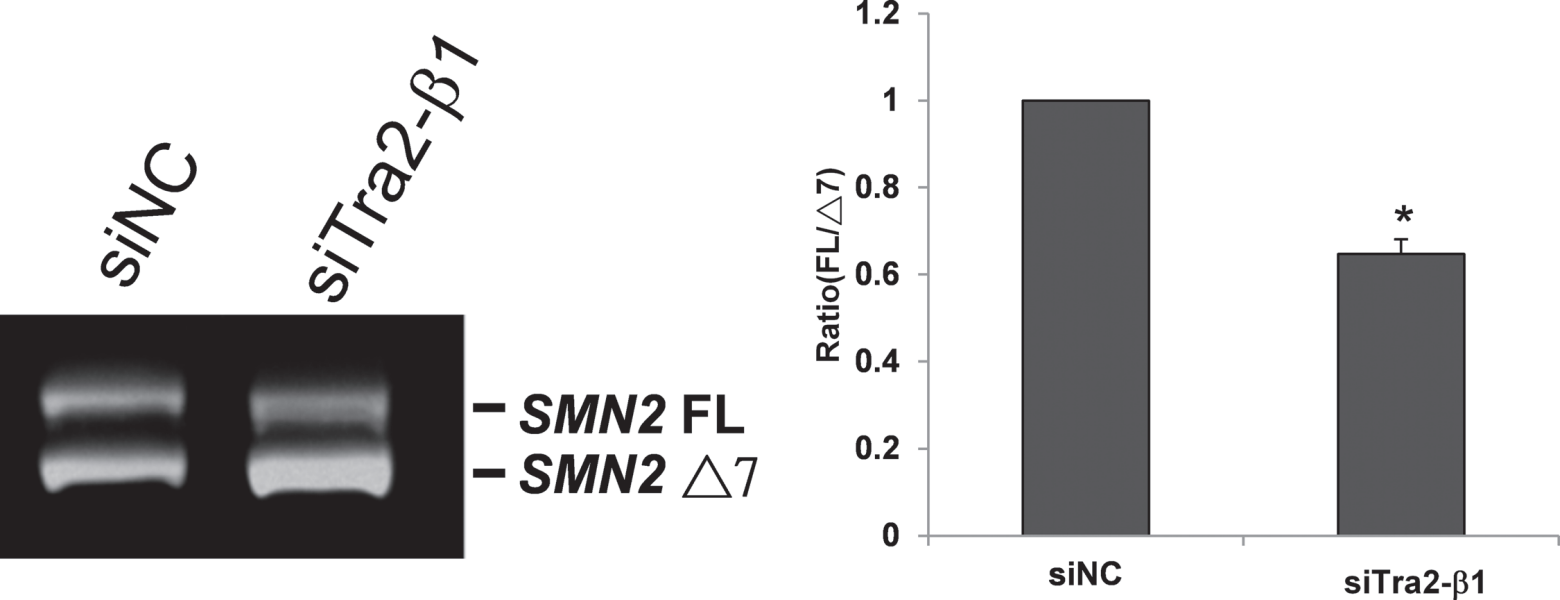
Knockdown of Tra2-β1 decreases *SMN2* exon 7 inclusion in primary testis cells of SMA mice. Primary testis cells of SMA mice were cultured for 72 hours and then transfected with Tra2-β1 siRNA or negative control (NC) siRNA for 48 hours. Total RNA was isolated from transfected cells and then subjected to RT-PCR to amplify *SMN2* FL and Δ7 mRNAs. The results show that knockdown of Tra2-β1 promoted *SMN2* exon 7 exclusion in primary testis cells of SMA mice. Error bars represent standard deviation. (*) *P* < 0.05, compared with the siNC control.

To further prove the role of Tra2-β1 in testis *SMN2* exon 7 splicing, we knocked down Tra2-β1 expression by siRNA in primary testis cells of SMA mice. To achieve a higher transfection efficiency, we cultured the primary testis cells for 72 hours to allow cell attachment and then transfected them with Tra2-β1 siRNA or negative control (NC) siRNA. Although the level of Tra2-β1 has decreased in 72-hour cultured cells, further knockdown of Tra2-β1 and comparison of its effects with NC siRNA can still allow us to prove the role of Tra2-β1 in testis *SMN2* exon 7 splicing. Knockdown of *Tra2-β1* mRNA was verified by quantitative RT-PCR. Then, we found that siRNA knockdown of Tra2-β1 decreased *SMN2* exon 7 inclusion compared with NC siRNA (Fig. 6), indicating that downregulation of Tra2-β1 promotes *SMN2* exon 7 exclusion in primary testis cells of SMA mice. In conclusion, our results indicate that high expression level of Tra2-β1 is responsible for increased *SMN2* exon 7 inclusion in the testis of SMA mice.

## Discussion

We were surprised to find that the testis of SMA mice expresses high level of *SMN2* full-length mRNA and SMN protein. In this study, we investigated the mechanism responsible for this special *SMN2* splicing pattern in the testis of SMA mice. We found that the level of the splicing factor Tra2-β1 decreases during testis cell primary culture, which is correlated with *SMN2* full-length mRNA downregulation. Also, the testis of SMA mice expresses the highest level of Tra2-β1 among the many tissues examined. Furthermore, overexpression of Tra2-β1 in primary testis cells of SMA mice enhances *SMN2* exon 7 inclusion, whereas knockdown of Tra2-β1 reduces *SMN2* exon 7 inclusion. Therefore, our results indicate that high expression level of Tra2-β1 is responsible for increased *SMN2* exon 7 inclusion in the testis of SMA mice. Previous studies had already shown that Tra2-β1 promoted the inclusion of *SMN* exon 7 and stimulated full-length *SMN2* expression [11]. However, these studies used cell lines, such as human HEK-293 and mouse NIH3T3, to demonstrate the effect of Tra2-β1. In contrast, our study is the first one to show the important role of Tra2-β1 in *SMN2* exon 7 splicing in an animal model. It remains to be shown whether this phenomenon also occurs in SMA patients.

In this study, we also found that overexpression of Tra2-β1 enhances *SMN2* mRNA exon 7 inclusion not only in primary testis cells but also in spinal cord neurons of SMA mice. The result suggests that the expression level of Tra2-β1 may be a modifying factor of SMA disease. In support of this hypothesis, Helmken *et al*. showed that Tra2-β1 protein is differentially expressed between type I-III SMA patients and between affected and unaffected siblings of discordant SMA families, at least in lymphoblastoid cells [36]. Differential expression of Tra2-β1 may account for the different ratios of *SMN2* full-length vs. truncated mRNA in type I-III SMA patients, which cannot be explained by different copy numbers of *SMN2* gene. An analysis of 36 SMA patients and their siblings from 15 discordant families did not identify any mutation or polymorphism within the coding and promoter regions of *Tra2-β1* gene [37], excluding a change on genomic level as a direct cause for differential expression of Tra2-β1 protein. Therefore, further studies on the regulatory mechanism of Tra2-β1 expression, especially in motor neurons, may reveal other SMA modifying factors that modulate full-length SMN production through regulating Tra2-β1 expression level.

Differential regulation of Tra2-β1 in testis tissue, primary testis cells, and testis cell lines is also intriguing. Identifying the mechanisms underlying this regulation may provide a way of modulating Tra2-β1 levels in motor neurons, and thus serve as a means of augmenting SMN. Previously, we suspected that high expression of Tra2-β1 in testis tissue might be induced by factors in testis microenvironment, which were not reproduced in cell culture. Thus, we treated testis cell lines with several testis microenvironmental factors, including low temperature, lactic acid, and androgens. Although we did not measure the Tra2-β1 levels directly, we found that these treatments could not increase *SMN2* exon 7 inclusion in testis cell lines. The mechanism of Tra2-β1 regulation in the testis requires further investigation.

A variety of potential therapies have been developed for SMA, including small molecule drugs, antisense oligonucleotides, polypeptides and proteins, gene therapy, and stem cell therapy [38]. One kind of small molecules is histone deacetylase (HDAC) inhibitor, such as valproic acid, phenylbutyrate, trichostatin A and suberoylanilide hydroxamic acid. Previous studies showed that trichostatin A (TSA) can promote *SMN2* gene transcription and increase survival rate in the SMA mouse model [39]. The mechanism of TSA activation of *SMN2* gene transcription is to inhibit histone deacetylation at the promoter. However, the effects of TSA on inhibiting histone deacetylation and activating transcription are not specific to *SMN2* gene. We have found that TSA can also activate *Tra2-β1* gene transcription and increase *SMN2* exon 7 inclusion (data not shown). In addition, we showed that overexpression of Tra2-β1, but not ASF/SF2, increases *SMN2* exon 7 inclusion in spinal cord neurons of SMA mice. Therefore, our results in this study suggest that Tra2-β1 may be a potential target for SMA therapy.

## Supporting Information

S1 Checklist The ARRIVE guidelines checklist for animal research. (DOCX)

S1 Fig Quantitative RT-PCR of *SMN2* mRNA in tissues of SMA mice. Various tissues of type 3 SMA mice were collected. Total RNA was isolated and subjected to quantitative real-time RT-PCR to amplify *SMN2* full-length (FL) and exon 7-lacking (Δ7) mRNAs independently. The expression levels of *SMN2* FL and Δ7 mRNA were normalized to GAPDH separately. Then, the FL/Δ7 ratios of various tissues were shown. The result showed that the testis expressed high level of *SMN2* FL mRNA.
(TIF)

S2 Fig Analysis of *SMN2* exon 7 splicing in testis-derived cell lines. Mouse testis cell lines, GC-1 spermatogonia, TM3 Leydig cells, and TM4 Sertoli cells, were transfected with the *SMN2* minigene plasmid for 48 hours. For comparison, mouse NSC34 motor neuron cell lines were also transfected with *SMN1* and *SMN2* minigene plasmids. Total RNA was isolated and subjected to RT-PCR to amplify *SMN1* and *SMN2* minigene FL and Δ7 mRNAs. The result showed that *SMN2* exon 7 inclusion was not promoted in these testis cell lines.
(TIF)

S3 Fig Quantitative RT-PCR of *SMN2* mRNA in primary testis cells of SMA mice. Total RNA was isolated from primary testis cells cultured for different time periods (2 hours, 48 hours, 96 hours) and subjected to quantitative real-time RT-PCR to amplify *SMN2* full-length (FL) and exon 7-lacking (Δ7) mRNAs independently. The expression levels of *SMN2* FL and Δ7 mRNA were normalized to GAPDH separately. Then, the FL/Δ7 ratios were shown. The result showed that *SMN2* FL mRNA was high in 2-hour cultured cells and decreased in 96-hour cultured cells.
(TIF)
