# Supplementary material for: High Expression Level of Tra2-β1 Is Responsible for Increased SMN2 Exon 7 Inclusion in the Testis of SMA Mice
Source: PLoS One. 2015 Mar 17;10(3):e0120721. doi: 10.1371/journal.pone.0120721 (PMC4363149; doi:10.1371/journal.pone.0120721)
Supplement: S1 Checklist — (DOCX) [file pone.0120721.s001.docx]

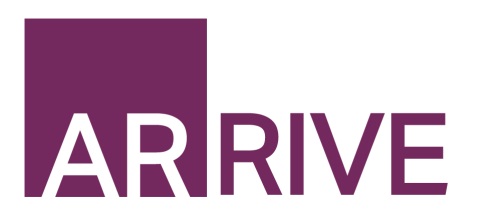


The ARRIVE Guidelines Checklist

Animal Research: Reporting In Vivo Experiments

Carol Kilkenny^1^, William J Browne^2^, Innes C Cuthill^3^, Michael Emerson^4^ and Douglas G Altman^5^

*^1^The National Centre for the Replacement, Refinement and Reduction of Animals in Research, London, UK, ^2^School of Veterinary Science, University of Bristol, Bristol, UK, ^3^School of Biological Sciences, University of Bristol, Bristol, UK, ^4^National Heart and Lung Institute, Imperial College London, UK, ^5^Centre for Statistics in Medicine, University of Oxford, Oxford, UK.*

|  | | ITEM | RECOMMENDATION | Section/ Paragraph |
| --- | --- | --- | --- | --- |
| 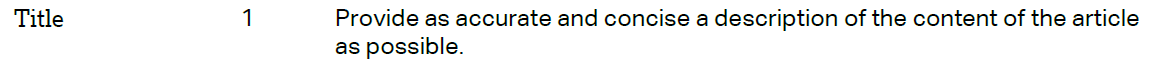 | | | High Expression Level of Tra2-beta1 Is Responsible for Increased SMN2 Exon 7 Inclusion in the Testis of SMA Mice |  |
| 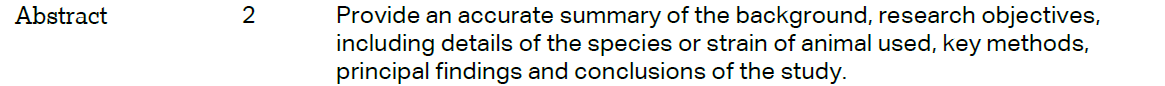 | | | Spinal muscular atrophy (SMA) is an inherited neuromuscular disease caused by deletion or mutation of *SMN1* gene. All SMA patients carry a nearly identical *SMN2* gene, which produces low level of SMN protein due to mRNA exon 7 exclusion. Previously, we found that the testis of C57BL/6 SMA mice (*smn*^−/−^ *SMN2*) expresses high level of *SMN2* full-length mRNA, indicating a testis-specific mechanism for *SMN2* exon 7 inclusion. To elucidate the underlying mechanism, we established primary cultures of testis cells from SMA mice and analyzed them for *SMN2* exon 7 splicing. We found that primary testis cells after a 2-hour culture still expressed high level of *SMN2* full-length mRNA, but the level decreased after longer cultures. We then compared the protein levels of relevant splicing factors, and found that the level of Tra2-β1 also decreased during testis cell culture, correlated with *SMN2* full-length mRNA downregulation. In addition, the testis of SMA mice expressed the highest level of Tra2-β1 among the many tissues examined. Furthermore, overexpression of Tra2-β1, but not ASF/SF2, increased *SMN2* minigene exon 7 inclusion in primary testis cells and spinal cord neurons of SMA mice. Therefore, our results indicate that high expression level of Tra2-β1 is responsible for increased *SMN2* exon 7 inclusion in the testis of SMA mice. This study also suggests that the expression level of Tra2-β1 may be a modifying factor of SMA disease and a potential target for SMA treatment. |  |
| INTRODUCTION | | |  |  |
| 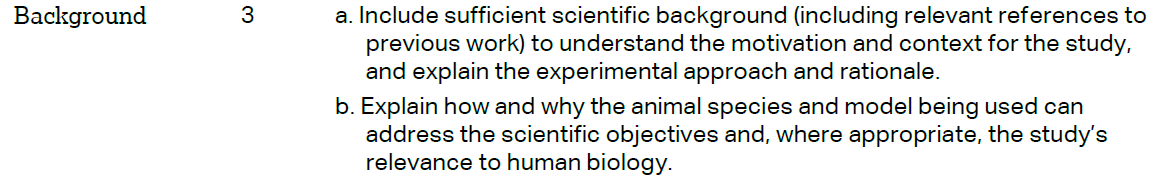 | | | Spinal muscular atrophy (SMA) is an autosomal recessive disease characterized by the loss of lower motor neurons and atrophy of muscles. According to age of onset and its severity, SMA is categorized into three main types (type I – III) and two subtypes (type 0 and type IV) [[1](#_ENREF_1),[2](#_ENREF_2)]. SMA occurs in approximately 1 in 6,000 to 10,000 newborns and has a carrier frequency of 1 in 35 to 50. SMA is the second most common autosomal recessive disease and the most common genetic cause of infant mortality [[3](#_ENREF_3)]. SMA is caused by deletions or mutations of *survival motor neuron 1* (*SMN1*) gene. All SMA patients carry a nearly identical *SMN2* gene. However, *SMN2* expresses only limited amount of functional SMN protein and is unable to compensate for the *SMN1* gene defect [[4](#_ENREF_4)]. The two genes differ by only five nucleotides, and the most important difference between *SMN1* gene and *SMN2* gene is a C-to-T transition at 6^th^ nucleotide of exon 7. The nucleotide transition causes exon 7 exclusion in most *SMN2* mRNA [[5-8](#_ENREF_5)]. The first SMA-like mouse models were developed independently at the same time by Li group and Burghes group [9,10]. Our previous study showed that the testis expressed high levels of *SMN2* full-length mRNA and SMN protein, which is different from other tissues of SMA mice. The mechanism of *SMN2* mRNA splicing in the testis of SMA mice needs further investigation.  To further investigate the mechanism of *SMN2* mRNA splicing in the testis of SMA mice, we established the primary testis cell culture from SMA-like mice. The SMA-like mice were generated by crossing *smn*^+/-^ mice with human *SMN2* transgene, which showed motor neuron degenerative symptoms [9]. |  |
| 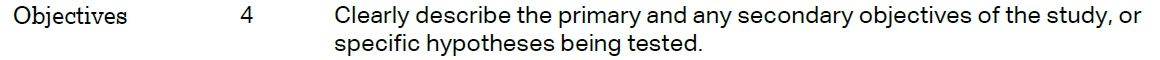 | | | The objectives of this study were to investigate the mechanism of *SMN2* mRNA splicing in the testis of SMA mice and the role of the mechanism in neuron cells of SMA mice. |  |
| METHODS | | |  |  |
| 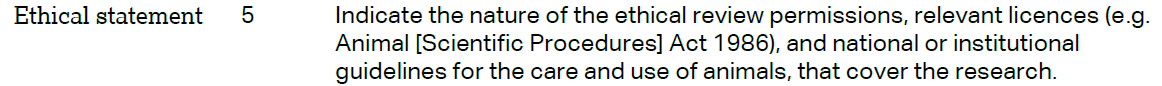 | | | All breeding and subsequent use of animals, including mice sacrifice, in this study were approved by the Institutional Animal Care and Use Committee of Kaohsiung Medical University. The IACUC approval number is 98033. |  |
| 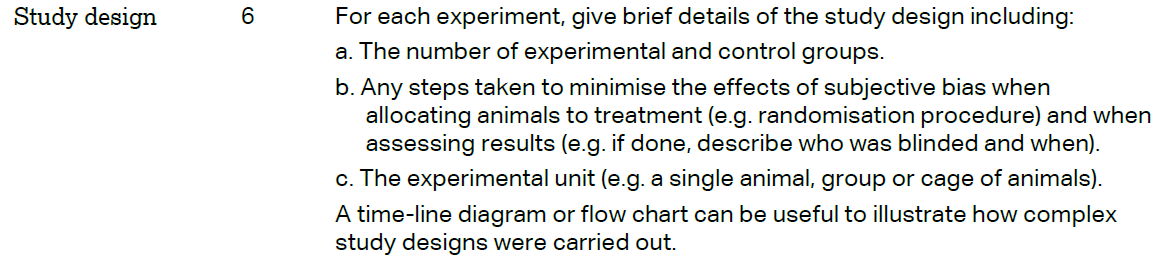 | | | 18-20 week old type 3 SMA mice were used in every experiment. The protein and mRNA expression levels were detected in six to eight independent SMA mice. The primary testis cells cultured for different time period were harvested from same mice and were repeated in three independent SMA mice. The primary neuron cells of spinal cord were harvested from E17.5 embryos of SMA mice (*smn^−/−^ SMN2^+^*). Six to eight embryos from the same litter were collected together. |  |
| 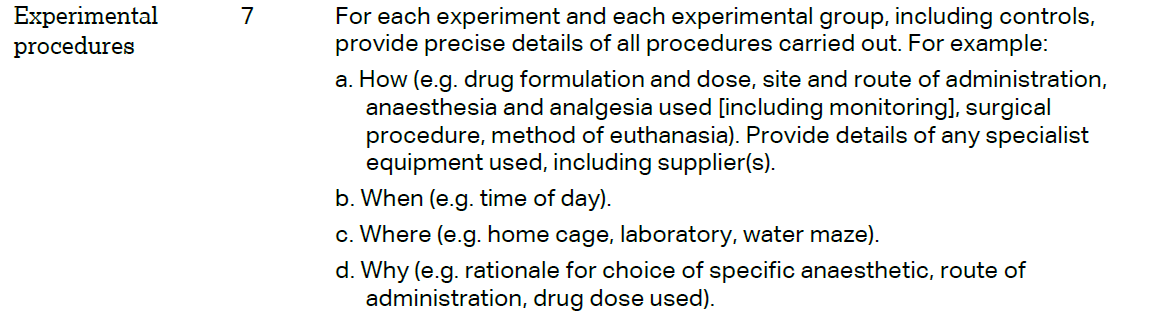 | | | 18-20 week old type 3 SMA mice were used in every experiment. All mice were sacrificed by high concentration of carbon dioxide and the tissues were harvested after sacrifice. |  |
| 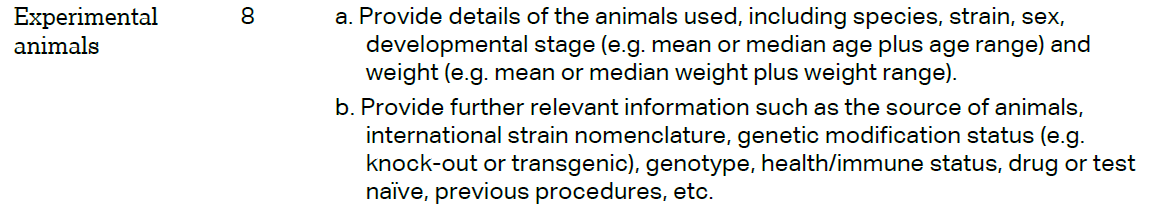 | | | The strain of SMA mice we used is B6;Cg-Tg(SMN2)Hung Smn1. The age of the SMA mice we used are 18-20 week old male and female mice for different experiments. |  |

The ARRIVE guidelines. Originally published in *PLoS Biology*, June 2010^1^

| 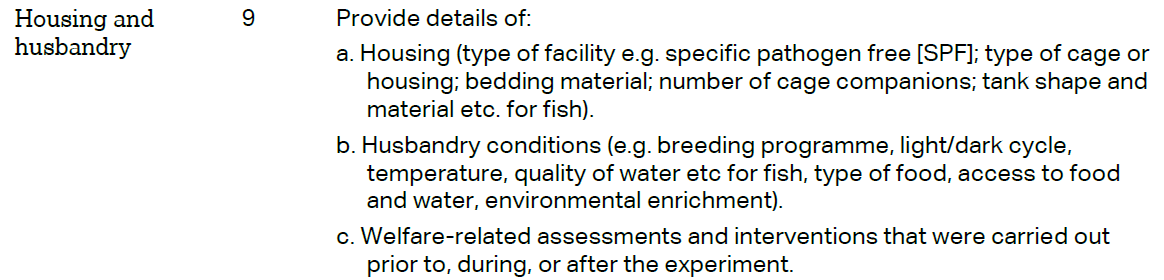 | All mice were housed with an inverse 12 hours day-night cycle in a temperature (22±2℃) controlled room. All mice were allowed free access to water and diet. The cages contained wood shavings, bedding and a cardboard tube for environmental enrichment. |  |
| --- | --- | --- |
| 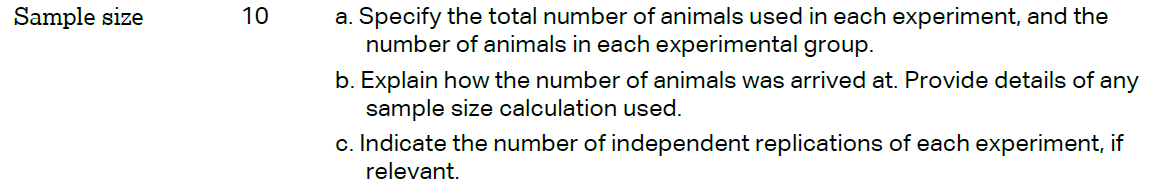 | The protein and mRNA expression levels were detected in six to eight independent SMA mice. The primary testis cells cultured for different time period were harvested from same mice and were repeated in three independent SMA mice. The total number of SMA mice we used is about 40. |  |
| 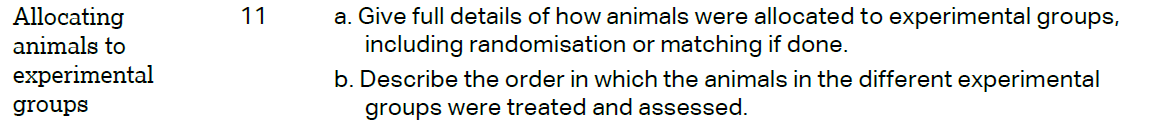 | The mice did not be allocated to groups in every experiment and were chosen randomly. |  |
| 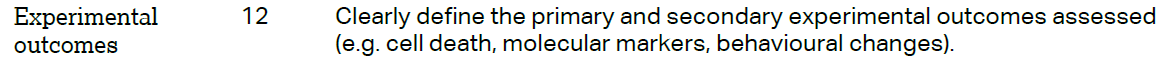 | The primary outcome was to analyze the SMN2 mRNA splicing forms in different tissues of SMA mice. The secondary outcomes were to analyze the SMN2 mRNA splicing forms and relevant splicing factor expressions in primary testis cells cultured for different time period. |  |
| 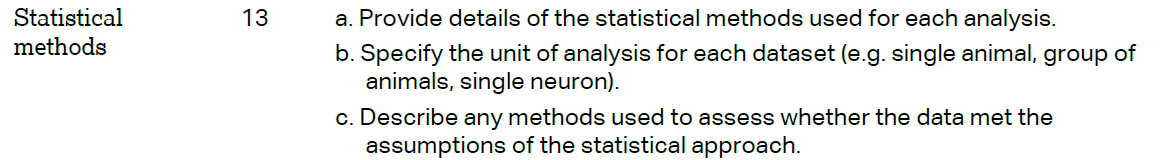 | Student’s *t* test was conducted to evaluate differences between groups. A probability of less than 0.05 was considered to be statistically significant. Error bars represent standard deviation. For each test, the experimental unit was an individual animal. |  |
| RESULTS |  |  |
| 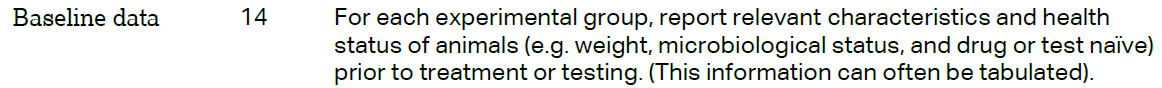 | The mice were free of 10 viral pathogens, 5 bacterial pathogens and 4 parasitic pathogens. The tests were done by National Laboratory Animal Center.   \| Pneumonia virus of mice (PVM) \| Reovirus Type 3 (Reo-3) \| \| --- \| --- \| \| Theiler’s murine encephalomyelitis virus (GD7) \| Sendai virus \| \| Minute virus of mice (MVM) \| Mouse hepatitis virus (MHV) \| \| Ectromelia virus (Pox virus) \| Mouse Parvovirus (MPV) \| \| *Mycoplasma pulmonis (M. pul)* \| Murine Norovirus (MNV) \| \| *Bordetella bronchiseptica* \| *Pseudomonas aeruginosa* \| \| *Citrobacter rodentium* \| *Salmonella spp.* \| \| *Helicobacter spp.* \| *Aspicularis tetraptera* \| \| *Hymenolepis diminuta* \| *Rodentolepis nana* \| \| Pinworm (Syphacia spp.) – cellophane tape \|  \| |  |
| 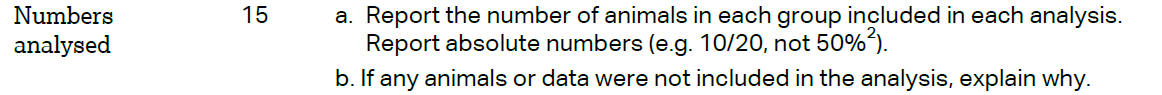 | The protein and mRNA expression levels were detected in six to eight independent SMA mice. The primary testis cells cultured for different time period were harvested from same mice and were repeated in three independent SMA mice. |  |
| 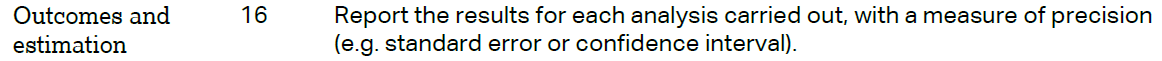 | Figure 5. Primary testis cells (A) and primary spinal cord neurons (B) of SMA mice were co-transfected with SMN2 minigene plasmid and Tra2-β1 overexpression plasmid, ASF/SF2 overexpression plasmid or blank vector as control for 48 hours. Total RNA was isolated from transfected cells and then subjected to RT-PCR to amplify SMN2 minigene FL and Δ7 mRNAs. The result showed that overexpression of Tra2-β1, but not ASF/SF2, remarkably increased SMN2 exon 7 inclusion in both primary testis cells and primary spinal cord neurons of SMA mice. Error bars represent standard deviation.  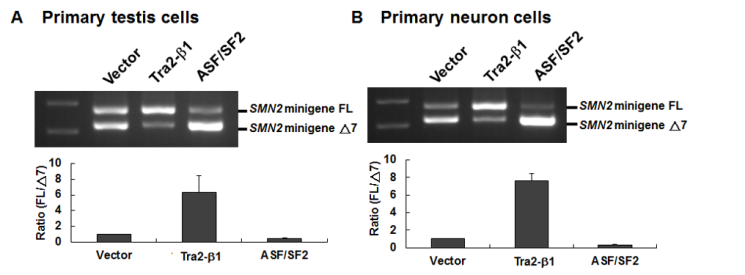 |  |
| 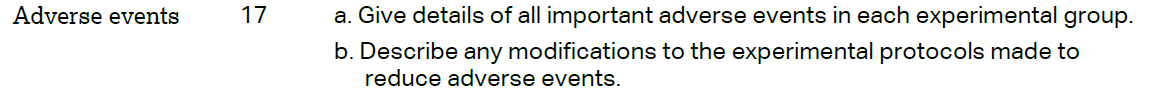 | There are no significant adverse events. |  |
| DISCUSSION |  |  |
| 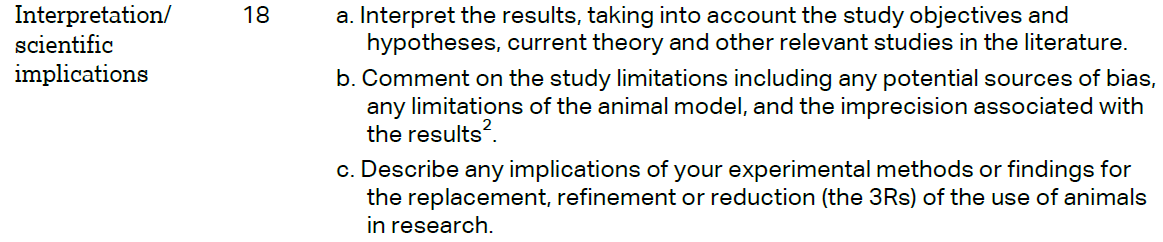 | We were surprised to find that the testis of SMA mice expresses high level of *SMN2* full-length mRNA and SMN protein. In this study, we investigated the mechanism responsible for this special *SMN2* splicing pattern in the testis of SMA mice. We found that the level of the splicing factor Tra2-β1 decreases during testis cell primary culture, which is correlated with *SMN2* full-length mRNA downregulation. Also, the testis of SMA mice expresses the highest level of Tra2-β1 among the many tissues examined. Furthermore, overexpression of Tra2-β1 in primary testis cells enhances *SMN2* exon 7 inclusion. Therefore, our results indicate that high expression level of Tra2-β1 is responsible for increased *SMN2* exon 7 inclusion in the testis of SMA mice. Previous studies had already shown that Tra2-β1 promoted the inclusion of *SMN* exon 7 and stimulated full-length *SMN2* expression [[11](#_ENREF_11)]. However, these studies used cell lines, such as human HEK-293 and mouse NIH3T3, to demonstrate the effect of Tra2-β1. In contrast, our study is the first one to show the important role of Tra2-β1 in *SMN2* exon 7 splicing in an animal model. It remains to be shown whether this phenomenon also occurs in SMA patients.  In this study, we also found that overexpression of Tra2-β1 enhances *SMN2* mRNA exon 7 inclusion not only in primary testis cells but also in spinal cord neurons of SMA mice. The result suggests that the expression level of Tra2-β1 may be a modifying factor of SMA disease. In support of this hypothesis, Helmken *et al.* showed that Tra2-β1 protein is differentially expressed between type I-III SMA patients and between affected and unaffected siblings of discordant SMA families, at least in lymphoblastoid cells [12]. Differential expression of Tra2-β1 may account for the different ratios of *SMN2* full-length vs. truncated mRNA in type I-III SMA patients, which cannot be explained by different copy numbers of *SMN2* gene. An analysis of 36 SMA patients and their siblings from 15 discordant families did not identify any mutation or polymorphism within the coding and promoter regions of *tra2-β1* gene [[13](#_ENREF_37)] , excluding a change on genomic level as a direct cause for differential expression of Tra2-β1 protein. Therefore, further studies on the regulatory mechanism of Tra2-β1 expression, especially in motor neurons, may reveal other SMA modifying factors that modulate full-length SMN production through regulating Tra2-β1 expression level. |  |
| 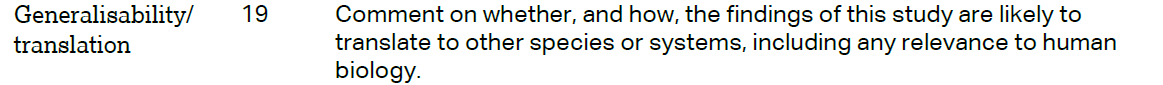 | We have showed that overexpression of Tra2-β1, but not ASF/SF2, increases *SMN2* exon 7 inclusion in primary testis cells and spinal cord neurons of SMA mice. In addition, we found that TSA can also activate *tra2-β1* gene transcription and increase *SMN2* exon 7 inclusion (data not shown). Therefore, our results in this study suggest that the expression level of Tra2-β1 may be a modifying factor of SMA disease and a potential target for SMA treatment. |  |
| 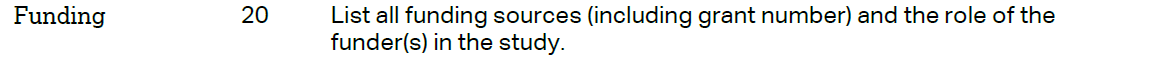 | | This study was supported by the Ministry of Science and Technology, Taiwan, R.O.C (NSC98-2320-B-037-025-MY3), (NSC98-2321-B-037-062-), (NSC99-2321-B-037-002-), (NSC100-2321-B-039-007-). |


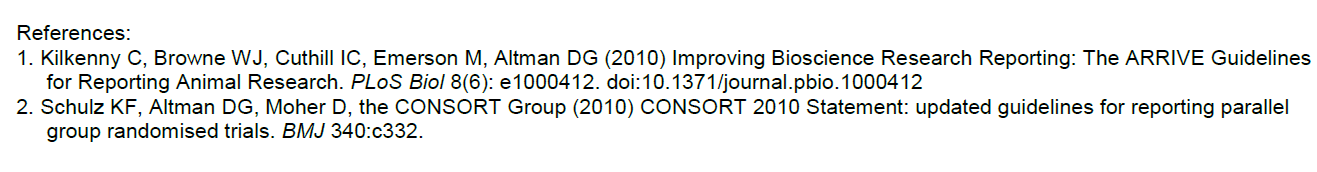


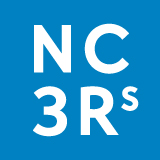
Reference

1. Kolb SJ, Kissel JT (2011) Spinal muscular atrophy: a timely review. Arch Neurol 68: 979-984.

2. Mercuri E, Bertini E, Iannaccone ST (2012) Childhood spinal muscular atrophy: controversies and challenges. Lancet Neurol 11: 443-452.

3. Ogino S, Leonard DG, Rennert H, Ewens WJ, Wilson RB (2002) Genetic risk assessment in carrier testing for spinal muscular atrophy. Am J Med Genet 110: 301-307.

4. Lefebvre S, Burglen L, Reboullet S, Clermont O, Burlet P, et al. (1995) Identification and characterization of a spinal muscular atrophy-determining gene. Cell 80: 155-165.

5. Nurputra DK, Lai PS, Harahap NI, Morikawa S, Yamamoto T, et al. (2013) Spinal muscular atrophy: from gene discovery to clinical trials. Ann Hum Genet 77: 435-463.

6. Burglen L, Lefebvre S, Clermont O, Burlet P, Viollet L, et al. (1996) Structure and organization of the human survival motor neurone (SMN) gene. Genomics 32: 479-482.

7. Lorson CL, Hahnen E, Androphy EJ, Wirth B (1999) A single nucleotide in the SMN gene regulates splicing and is responsible for spinal muscular atrophy. Proc Natl Acad Sci U S A 96: 6307-6311.

8. Monani UR, Lorson CL, Parsons DW, Prior TW, Androphy EJ, et al. (1999) A single nucleotide difference that alters splicing patterns distinguishes the SMA gene SMN1 from the copy gene SMN2. Hum Mol Genet 8: 1177-1183.

9. Hsieh-Li HM, Chang JG, Jong YJ, Wu MH, Wang NM, et al. (2000) A mouse model for spinal muscular atrophy. Nat Genet 24: 66-70.

10. Monani UR, Sendtner M, Coovert DD, Parsons DW, Andreassi C, et al. (2000) The human centromeric survival motor neuron gene (SMN2) rescues embryonic lethality in Smn(-/-) mice and results in a mouse with spinal muscular atrophy. Hum Mol Genet 9: 333-339.

11. Hofmann Y, Lorson CL, Stamm S, Androphy EJ, Wirth B (2000) Htra2-beta 1 stimulates an exonic splicing enhancer and can restore full-length SMN expression to survival motor neuron 2 (SMN2). Proc Natl Acad Sci U S A 97: 9618-9623.

12. Helmken C, Hofmann Y, Schoenen F, Oprea G, Raschke H, et al. (2003) Evidence for a modifying pathway in SMA discordant families: reduced SMN level decreases the amount of its interacting partners and Htra2-beta1. Hum Genet 114: 11-21.

13. Helmken C, Wirth B (2000) Exclusion of Htra2-beta1, an up-regulator of full-length SMN2 transcript, as a modifying gene for spinal muscular atrophy. Hum Genet 107: 554-558.
