# Supplementary figures and images for: High Expression Level of Tra2-β1 Is Responsible for Increased SMN2 Exon 7 Inclusion in the Testis of SMA Mice
Source: PLoS One. 2015 Mar 17;10(3):e0120721. doi: 10.1371/journal.pone.0120721 (PMC4363149; doi:10.1371/journal.pone.0120721)

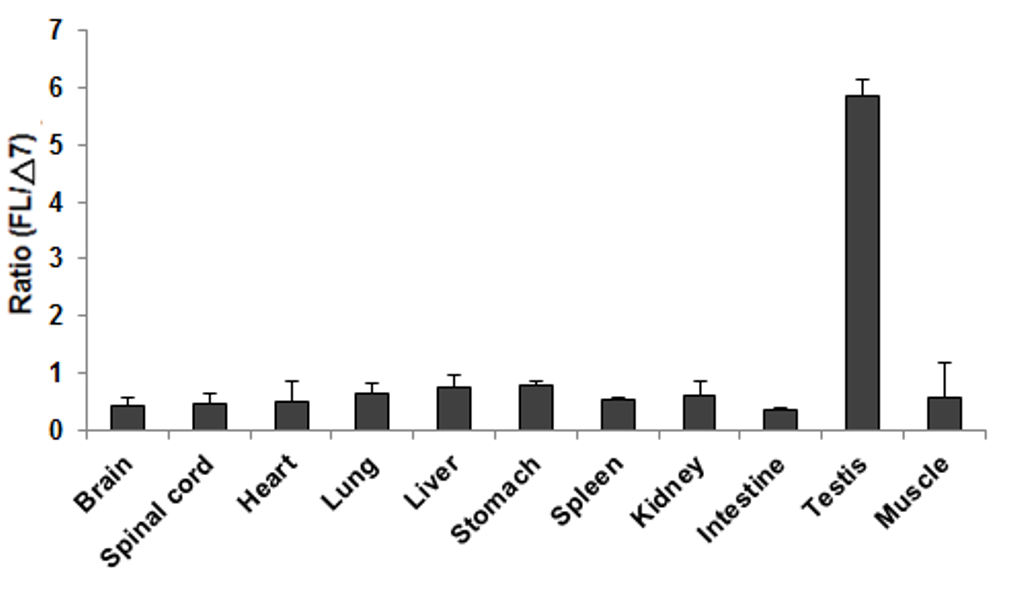

Supplement: S1 Fig — Various tissues of type 3 SMA mice were collected. Total RNA was isolated and subjected to quantitative real-time RT-PCR to amplify SMN2 full-length (FL) and exon 7-lacking (Δ7) mRNAs independently. The expression levels of SMN2 FL and Δ7 mRNA were normalized to GAPDH separately. Then, the FL/Δ7 ratios of various tissues were shown. The result showed that the testis expressed high level of SMN2 FL mRNA. (TIF) [file pone.0120721.s002.tif]

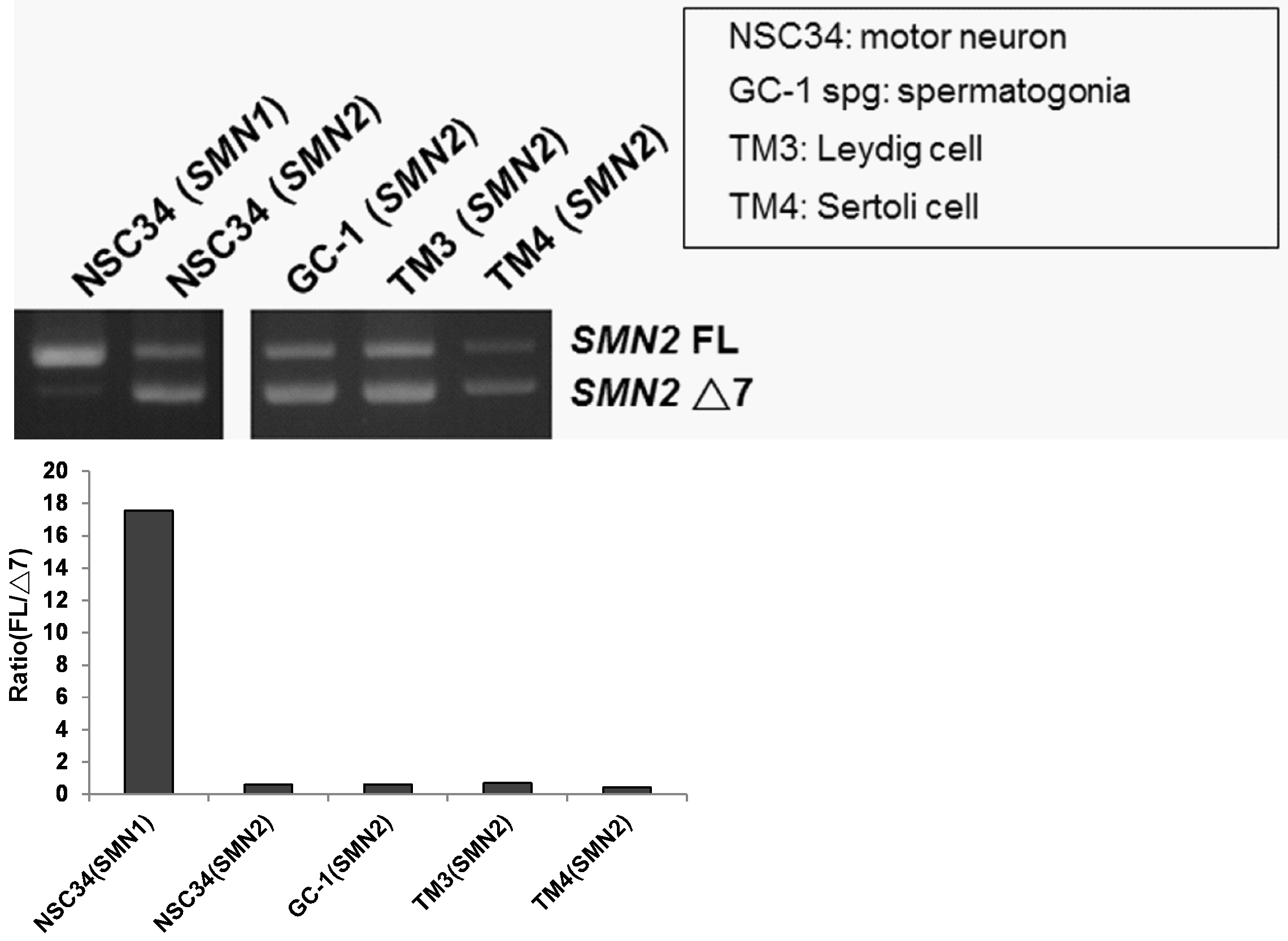

Supplement: S2 Fig — Mouse testis cell lines, GC-1 spermatogonia, TM3 Leydig cells, and TM4 Sertoli cells, were transfected with the SMN2 minigene plasmid for 48 hours. For comparison, mouse NSC34 motor neuron cell lines were also transfected with SMN1 and SMN2 minigene plasmids. Total RNA was isolated and subjected to RT-PCR to amplify SMN1 and SMN2 minigene FL and Δ7 mRNAs. The result showed that SMN2 exon 7 inclusion was not promoted in these testis cell lines. (TIF) [file pone.0120721.s003.tif]

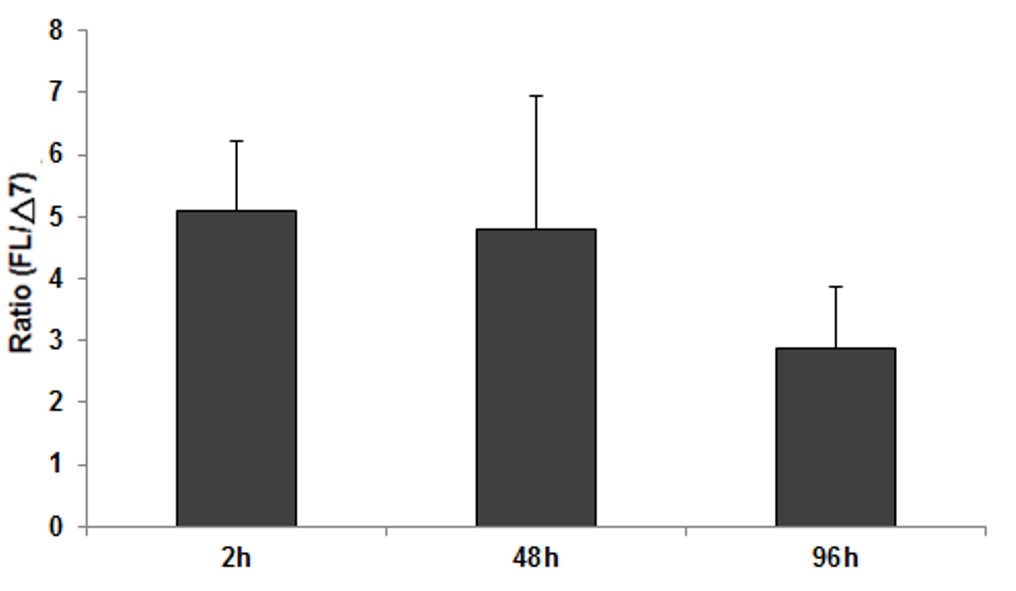

Supplement: S3 Fig — Total RNA was isolated from primary testis cells cultured for different time periods (2 hours, 48 hours, 96 hours) and subjected to quantitative real-time RT-PCR to amplify SMN2 full-length (FL) and exon 7-lacking (Δ7) mRNAs independently. The expression levels of SMN2 FL and Δ7 mRNA were normalized to GAPDH separately. Then, the FL/Δ7 ratios were shown. The result showed that SMN2 FL mRNA was high in 2-hour cultured cells and decreased in 96-hour cultured cells. (TIF) [file pone.0120721.s004.tif]
